# Supplementary material for: Disparities in Head and Neck Cancer: A Case for Chemoprevention with Vitamin D
Source: Nutrients. 2020 Aug 29;12(9):2638. doi: 10.3390/nu12092638 (PMC7551909; doi:10.3390/nu12092638)
Supplement: Supplementary file 1 [file nutrients-12-02638-s001.zip › Supplementary Materials-proof reading/Figure S1. Vitamin D3 inhibits HNC cell viability .docx]

**A**


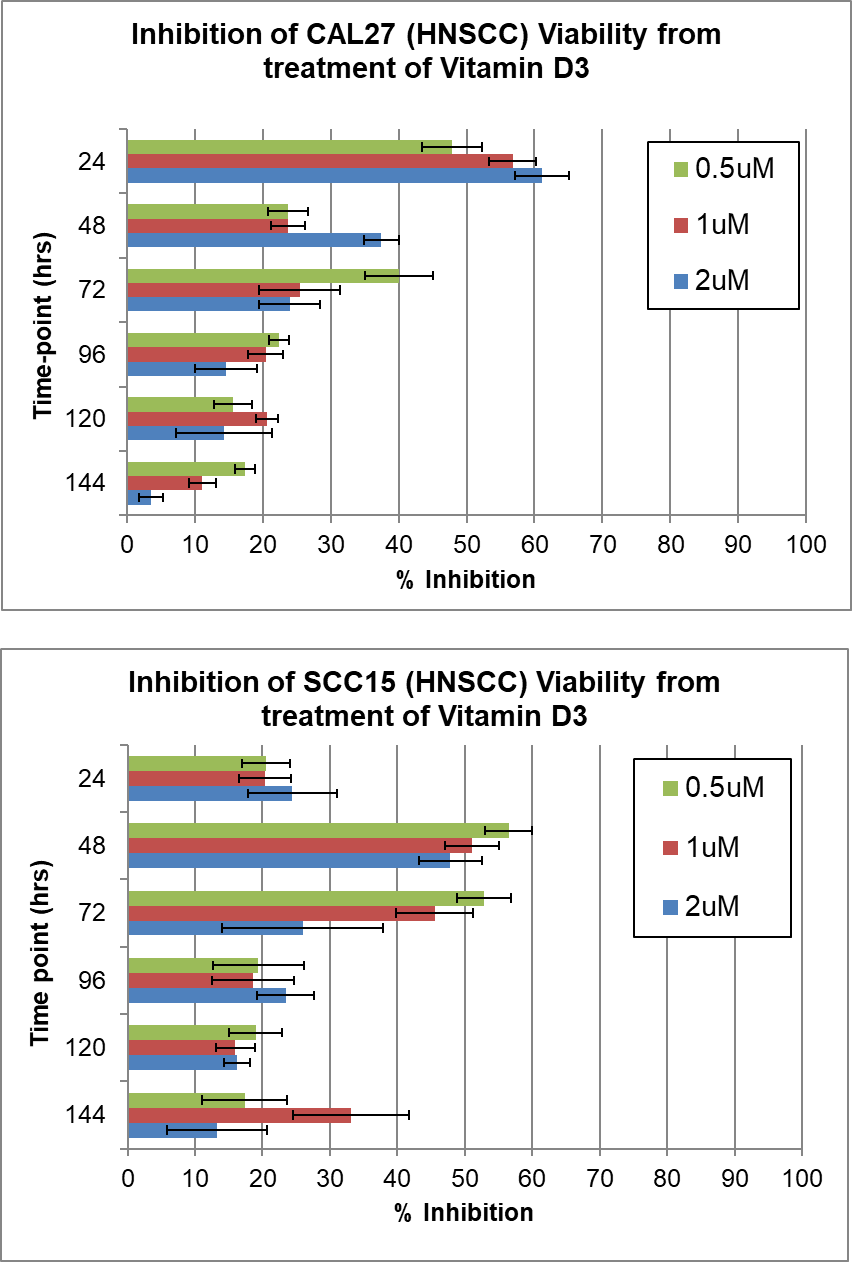


**B**

**Figure S1.** Vitamin D3 inhibits HNC cell viability. (**A**) CAL-27 (8E3 cells/well) and (**B**) SCC-15 (6E3 cells/well), cells were seeded into 96-well plates, allowed to adhere overnight and treated with 0.5, 1 or 2µM Vitamin D3. Viability was measured using the WST-1 viability assay following manufacturer’s instructions. Data are reported as Mean ± SEM of at least 6 wells per treatment per time point.
