# Supplementary material for: Disparities in Head and Neck Cancer: A Case for Chemoprevention with Vitamin D
Source: Nutrients. 2020 Aug 29;12(9):2638. doi: 10.3390/nu12092638 (PMC7551909; doi:10.3390/nu12092638)
Supplement: Supplementary file 1 [file nutrients-12-02638-s001.zip › Supplementary Materials-proof reading/Table S3. Validated gene targets altered in both CAL-27 and SCC-25 HNC cell lines by vitamin D (n=45).docx]

| **Table S3.** Validated gene targets altered in both CAL-27 and SCC-25 HNC cell lines by vitamin D (n=45) | | | | | | | | |
| --- | --- | --- | --- | --- | --- | --- | --- | --- |
| ATP7A | CACNA1A | CBX4 | CCDC141 | CD164 | COIL | COL4A1 | CPEB2 | DERL2 |
| DNAJC21 | EGFR | ENTHD1 | FAM84B | FOXF2 | H6PD | HMBOX1 | HSPA1B | IGF1R |
| ITGA1 | KCNJ2 | KLHL15 | LBR | LDLR | LIFR | MAMLD1 | MDM4 | NUFIP2 |
| PAK3 | PHF13 | PLEKHG4 | POLD3 | PTMA | QSER1 | RAB31 | RHOB | SAMD8 |
| SC5D | SOX5 | SPRED1 | SREBF1 | TMEM170B | TP53INP1 | UTP4 | VEGFA | ZNF460 |
