# Supplementary material for: Disparities in Head and Neck Cancer: A Case for Chemoprevention with Vitamin D
Source: Nutrients. 2020 Aug 29;12(9):2638. doi: 10.3390/nu12092638 (PMC7551909; doi:10.3390/nu12092638)
Supplement: Supplementary file 1 [file nutrients-12-02638-s001.zip › Supplementary Materials-proof reading/Table S4. Overlapping target genes of miRs altered in the same direction by vitamin D (n=32) .docx]

| **Table S4.** Overlapping target genes of miRs altered in the same direction by vitamin D (n=32) | | | | | |
| --- | --- | --- | --- | --- | --- |
| MicroRNA Alteration | Target Genes | | | | |
| Down-regulated *** | ATP7A | CBX4 | CCDC141 | ZNF460 | FAM84B |
|  | COL4A1 | CPEB2 | DNAJC21 | EGFR | DERL2 |
|  | IGF1R | HSPA1B | H6PD | ITGA1 | HMBOX1 |
|  | VEGFA | KLHL15 | LBR | LDLR | LIFR |
|  | MAMLD1 | UTP4 | TMEM170B | TP53INP1 | RHOB |
|  | SAMD8 | SC5D | SREBF1 | NUFIP2 | PAK3 |
|  | PHF13 | SPRED1 |  |  |  |
| Up-regulated # | POLD3 |  |  |  |  |
| *** Specific miRs down-regulated by vitamin D treatment, miR-7-1-3p and miR-335, with their listed overlapping gene targets (n=32); *#* specific miRs up-regulated by vitamin D treatment, miR-632 and miR-331-5p, with their only overlapping gene target (n=1), miR-616-3p is excluded from this table because it did not share any overlapping gene targets with the other up-regulated miRs. | | | | | |
